# The Predictive Value of Cystatin C in Monitoring of B Non-Hodgkin Lymphomas: Relation to Biochemical and Clinical Parameters

**DOI:** 10.1155/2013/752792

**Published:** 2013-09-16

**Authors:** A. Softić, L. Begić, A. Halilbašić, T. Vižin, J. Kos

**Affiliations:** ^1^Department of Biochemistry, Faculty of Pharmacy, University of Tuzla, 75000 Tuzla, Bosnia and Herzegovina; ^2^Clinic for Oncology, Haematology and Radiotherapy, University Clinical Centre Tuzla, 75000 Tuzla, Bosnia and Herzegovina; ^3^Chair of Pharmaceutical Biology, Faculty of Pharmacy, University of Ljubljana, 1000 Ljubljana, Slovenia; ^4^Department of Biotechnology, Jožef Stefan Institute, 1000 Ljubljana, Slovenia

## Abstract

The predictive value of cystatin C as a marker of course of the disease has been evaluated. Fifty-two pairs of serum samples of patients with B non-Hodgkin lymphoma have been collected at the time of diagnosis and before fourth cycle of chemotherapy. The levels of cystatin C, CRP, **β**_2_M, LDH, and IL-6 in samples have been measured, and clinical parameters of course of the disease (B symptoms, clinical stage, patients' age, and IPI) have been noted. In total patient's group cystatin C levels correlated with **β**_2_M and IPI. In aggressive lymphomas, the inhibitor levels correlated with clinical stage of disease and were significantly higher in patients with elevated LDH activity. In aggressive nodal lymphomas its levels correlated with **β**_2_M, IPI, and clinical stage of disease. The cystatin C level was significantly increased in total group of patients over 60 years old, while in particular types of lymphoma, no statistical significance has been obtained. Our results indicate that cystatin C should be taken into consideration in disease monitoring. However, we expect that the disease-free and overall survival analysis will give the definitive answer about the reliability of cystatin C as an indicator of course of aggressive lymphomas.

## 1. Introduction

Cysteine proteinase inhibitors, cystatins, are involved in mechanisms controlling intracellular and extracellular protein degradation. Under normal physiological conditions, small amounts of catalytically active proteases, released from lysosomes or secreted from infected or dying cells, are effectively blocked by cystatins. A disbalance between proteinases and their natural inhibitors leads to the development of various diseases. 

Cystatin C, a member of family II of cystatins, is a nonglycosylated 13 kDa protein inhibitor of cysteine proteases, with widespread distribution in almost all extracellular fluids, the highest levels having been determined in cerebrospinal fluid, seminal plasma, and synovial fluid [1]. 

A broad spectrum of biological roles has been suggested for cystatin C, including control of protein catabolism, regulation of hormone processing and bone resorption, inflammation, antigen presentation, and T-cell dependent immune response [2, 3]. 

Cystatin C was suggested as a marker of glomerular filtration rate, since it is produced at a constant rate by all nucleated body cells, freely filtered in the renal glomeruli and almost completely reabsorbed and catabolized in the proximal tubules [4]. 

Cystatin C has also been suggested as playing a role associated with alteration of the proteolytic system in cancer. In patients with colorectal, lung, and melanoma carcinomas high serum levels of cystatin C are associated with poor outcome of disease [5–7]. 

Thus, cystatin C level was proposed for followup of diseases, efficacy of chemotherapy, and prediction of disease outcome. Our recent study showed that patients with B non-Hodgkin lymphomas had significantly higher cystatin C levels compared to healthy controls. In addition, patients with relapse of the disease had significantly higher cystatin C level compared to patients without relapse [8]. 

For sake of evaluation of the predictive value of cystatin C as a marker of course of the disease, the cystatin C levels were compared with established biochemical parameters (C reactive protein (CRP), beta-2 microglobulin (*β*_2_M), lactate dehydrogenase (LDH) levels/activities), with clinical parameters of disease progression (the presence of B symptoms, clinical stage, age, and international prognostic index (IPI)), and with suggested prognostic factor in patients with lymphoma [9] (the levels of cytokine interleukin 6 (IL-6)). 

## 2. Materials and Methods

### 2.1. Subjects

Serum samples of patients with diagnosis of B non-Hodgkin lymphoma have been collected from February 2008 until May 2011 at the Clinic for Oncology, Hematology and Radiotherapy of the University Clinical Centre of Tuzla, with the approval of the local ethical committee and the written consent of the participants. Two samples of blood from each patient were taken, first at the time of diagnosis, before therapy, and the second immediately before fourth cycle of chemotherapy. Thus, it was possible to exclude the possibility of increasing the cystatin C sera level due to influence of corticosteroids, which are the common part of chemotherapeutic protocols [10–12]. 

From fifty-two pairs of collected samples, three pairs were from the patients with the relapse of the disease. Eleven patients had the indolent type of lymphoma, and 41 had the aggressive form, of those 18 with nodal and 23 with extranodal disease presentation. 

Thirty-six patients have been receiving CHOP regimen of chemotherapy (cyclophosphamide, doxorubicin hydrochloride, oncovin, and prednisone). Among them, thirty patients have been receiving Rituximab and one patient bleomycin. Eight patients have been receiving CHOEP protocol (CHOP + etoposide) and 6 of them with addition of immunotherapy. Three patients have been receiving COP protocol (cyclophosphamide, oncovin, and prednisone), 2 patients FC/D protocol (fludarabine, cyclophosphamide, and dexamethasone), one patient high doses of methotrexate, while 2 patients with highly aggressive lymphomas have been treated by modified Yugoslav protocol for acute lymphoblastic leukemia. These last two patients have been included in group of aggressive lymphomas. 

For each patient, his age, type of lymphoma, and IPI have been noted, and his biochemical parameters, like CRP, *β*_2_M, LDH, and creatinine in serum, have been measured.

The serum creatinine level has been used as a criterion for selection of patients; those with the creatinine level above the upper physiologic limit have not been included in the study because of possible renal dysfunction. Also, patients with autoimmune diseases, asthma, and other malignant diseases have been excluded from the study.

Seven milliliters of blood per patient has been collected. The blood was clotted at room temperature and subsequently centrifuged at 3000 rpm. Serum was separated, aliquoted, and stored at −20°C. For analysis delayed for a longer period of time, serum was stored at –80°C. 

Reevaluation was carried out after three cycles of chemotherapy by repeating the tests which had results out of normal range. A complete response was defined as the disappearance of all clinical, laboratory, and radiographic evidence of lymphoma. Responding patients with minimal residual radiographic abnormalities were classified as showing partial response. Lesser objective responses were classified as poor partial response. 

### 2.2. Methods

The levels of cystatin C in sera of lymphoma patients were measured by specific sandwich-type ELISA, developed at the Department of Biochemistry and Molecular Biology, Jožef Stefan Institute and Krka, d.d., as described [7]. A microtiter plate was coated with rabbit affinity purified anti-cystatin C polyclonal antibody. Recombinant human cystatin C was used for preparation of calibration curves. Sera from patients were diluted 1 : 100 prior to being applied to wells of microtiter plate. As a detection antibody, 1A2 monoclonal antibody was used, conjugated to horseradish peroxidase. A substrate for peroxidase was 3,3′,5,5′-tetramethylbenzidine liquid substrate system (TMB, Sigma). The reaction was stopped by adding of 2 M H_2_SO_4_, and the absorbance was measured at 450 nm on minireader (TECAN, Sunrise). Measured values of diluted samples and controls were compared with the calibration curve and expressed in ng/mL of serum. 

The levels of IL-6 in sera of lymphoma patients were measured by commercial sandwich-type ELISA (Quantikine R&D Systems Inc. Minneapolis, USA) on minireader (TECAN, Sunrise). ELISA assays were performed in duplicate and the mean value determined. 

Biochemical parameters were measured at the Polyclinic for Laboratory Diagnostics, University Clinical Centre Tuzla. CRP was measured by immunoturbidimetric method on Architect c8000 analyzer. *β*_2_M was measured by Microparticle Enzyme Immunoassay (MEIA) (Abbott) on AxSYM system. LDH was measured by enzyme method (Abbott) on Architect c8000 analyzer and enzyme method (Siemens) on Dimension system. Creatinine was measured by a modified Jaffe method on Dimension system. 

### 2.3. Statistical Analysis

For statistical analysis of the results SPSS software, release 17.0. (SPSS Inc., Chicago, IL, USA) was used. Mann-Whitney *U* test was used to compare differences in cystatin C levels between independent groups, and the Wilcoxon matched pair test was used to compare cystatin C level measured at the time of diagnosis and before fourth cycle of chemotherapy. Correlations of cystatin C with clinical stage of disease, IL-6, CRP, *β*_2_M, and IPI were defined by Spearman rank correlation analysis. In all tests, two-sided *P*s below 0.05 were considered significant. 

## 3. Results

In patients who achieved partial or complete remission after three cycles of chemotherapy, the level of cystatin C significantly decreased, in total patient's group and in group with aggressive lymphoma (Table 1).

Out of 40 patients with disease remission, twenty-four (60%) patients had lower cystatin C level before fourth cycle of chemotherapy compared to the level measured at the time of diagnosis. In the group of aggressive lymphomas (*N* = 34), twenty-one (61,8%) patients had the lower cystatin C level before fourth cycle of therapy.

Out of total patient's number (*N* = 52), IL-6 was detected in 31 patients at the time of diagnosis (median: 5,29 pg/mL; range: 1,75–92,69 pg/mL). In the group of aggressive lymphomas, it was detected in 26 patients (median: 5,25 pg/mL; range: 1,75–92,69 pg/mL), in 13 patients with extranodal (median: 4,86 pg/mL; range: 3,32–25,74 pg/mL) and in 13 patients with nodal disease presentation (median: 5,33 pg/mL; range: 1,75–92,69 pg/mL). The results of correlation between cystatin C and IL-6 are presented in Table 2. In all groups of patients these two parameters showed no correlation. 

Before fourth cycle of chemotherapy, IL-6 was detected in 26 patients (median: 5,95 pg/mL; range: 0,88–25,73 pg/mL). In the group of aggressive lymphomas, it was detected in 20 patients (median: 5,39 pg/mL; range: 1,88–25,73 pg/mL), in 11 patients with extranodal (median: 5,01 pg/mL; range: 3,25–25,73 pg/mL) and in 9 patients with nodal disease presentation (median: 6,49 pg/mL; range: 1,88–23,17 pg/mL). The results of correlation between cystatin C and IL-6 before fourth cycle of chemotherapy are presented in Table 2. In all groups of patients these two parameters showed no correlation. 

At the time of diagnosis, the median level of CRP in total patient's group was 7,60 mg/L (range: 0,30–254,50 mg/L), in the group of aggressive lymphomas CRP was 8,80 mg/L (range: 0,80–254,50 mg/L), in the group with extranodal disease presentation CRP was 7,70 mg/L (range: 0,80–78,80 mg/L), and in the group with nodal disease presentation CRP was 13,65 mg/L (range: 1,90–254,50 mg/L). The cystatin C sera levels were compared with the CRP levels at the time of diagnosis (Table 2). In all groups of patients these two parameters showed no correlation. 

At the time of diagnosis, the median level of *β*_2_M in total patient's group was 1,86 mg/L (range: 0,51–4,25 mg/L), in the group of aggressive lymphomas *β*_2_M was 1,82 mg/L (range: 0,51–4,25 mg/L), in the group with extranodal disease presentation *β*_2_M was 1,67 mg/L (range: 0,51–3,85 mg/L), and in the group with nodal disease presentation *β*_2_M was 1,89 mg/L (range: 1,13–4,25 mg/L). 

In patients with partial or complete remission after three cycles of chemotherapy, the median *β*_2_M was in the total patient's group 1,8 mg/L (range: 1,0–4,29), in the group of aggressive lymphomas 1,83 mg/L (range: 1,0–4,29 mg/L), in the group with extranodal disease presentation 1,85 mg/L (range: 1,0–4,29 mg/L), and in the group with nodal disease presentation 1,72 mg/L (range: 1,10–3,98 mg/L). 

At the time of diagnosis, cystatin C showed significant correlation with *β*_2_M in total group of patients and in the group of nodal aggressive lymphomas (Table 2). Considering the obtained correlations and the fact that the increased levels of *β*_2_M indicate the patients with the aggressive lymphomas who will experience relapse [13], we analyzed correlation between cystatin C and *β*_2_M in patients with partial or complete remission after three cycles of chemotherapy (Table 2). Significant correlation was obtained in total group of patients with disease remission and in group with aggressive nodal lymphoma. In patients who did not experience disease remission after three cycles of chemotherapy, correlation between cystatin C and *β*_2_M was not calculated because of small number of patients per group. 

For the measurement of LDH two methods were used, and it was not possible to correlate LDH and cystatin C levels. Instead, patients were divided in two groups, a group with normal and a group with elevated LDH level (Table 3). Then, the cystatin C values of these two groups were compared, for each type of lymphoma. In patients with aggressive lymphoma, the group with elevated LDH had significantly higher level of cystatin C than the group with normal LDH activity. 

Based on the presence or absence of B symptoms (weight loss over 10% for a six month, night sweats, fever, and unexplained itching) patients were divided in two groups, with and without symptoms, for each type of lymphoma. Then, the values of cystatin C of these two groups were compared (Table 3). In all types of lymphoma no statistical significant difference was obtained. 

Based on the age, patients were divided in two groups, ≥60 years and <60 years old (Table 3). Then, the values of cystatin C of these two groups were compared, for each type of lymphoma. In total patients group, those ≥60 years old had significantly higher inhibitor level than the patients <60 years old.

The correlation between cystatin C and clinical stage of disease at the time of diagnosis was significant in the group of aggressive lymphomas and in subgroup with nodal presentation (Table 4). 

The correlation between cystatin C and IPI at the time of diagnosis was significant in total group of patients and in the group with nodal aggressive lymphomas (Table 4). 

## 4. Discussion

Our previous study showed that the level of cystatin C was significantly higher in patients who experienced relapse compared to patients newly diagnosed with non-Hodgkin lymphomas. Thus, we assumed that the level of this inhibitor can serve as a marker of relapse. In this study's framework it was not possible to reach the minimal number of patients for statistical analysis, with sample taken at diagnosis of disease, in clinical remission, and at diagnosis of relapse, because such approach would require the inclusion of several clinical centers, numerous researchers, and years of research. For this reason, we evaluated the predictive value of cystatin C in disease monitoring in two ways. 

The first method involved the separation of group of patients who were in partial or complete remission before admission of fourth cycle of chemotherapy. In this group then, we compared the levels of the inhibitor measured at the time of diagnosis with the levels measured before fourth cycle of chemotherapy. It was found that the level of inhibitor significantly decreases with the disease remission. Aggressive lymphomas almost completely contributed to statistical significance of complete sample (Table 1). Does this mean that there is a specific mechanism in aggressive lymphomas, not present in patients with indolent lymphoma, which contributes to decreased levels of cystatin C in disease remission? It could not be precisely determined due to the small sample of patients with indolent lymphomas (*N* = 6) who get into remission after the third cycle of chemotherapy. 

Another way to test the predictive value of cystatin C in disease monitoring was to compare its level with established biochemical (CRP, *β*_2_M, and LDH levels), clinical parameters of disease progression (the presence of B symptoms, clinical stage, age, and IPI) and with the levels of IL-6, as prognostic factor in patients with lymphoma [9]. 

Since the *β*_2_M is a light chain of antigens A, B and C of the main histocompatibility complex, the rate at which it is released into the circulation is determined by the activation and proliferation of lymphocytes [14]. Previous studies have shown that *β*_2_M correlated with the clinical stage of the disease in non-Hodgkin lymphomas [15, 16]. Johnson and colleagues [17] found that the outcome of chemotherapy in patients with aggressive non-Hodgkin lymphomas correlates with initial values of *β*_2_M: in the group with normal values, 70% of patients reached a clinical remission, and in the group of patients with elevated *β*_2_M, only 37% of patients achieved a clinical remission (*P* < 0.001). In addition, patients with normal initial *β*_2_M values had significantly longer period of clinical remission compared to patients with elevated *β*_2_M (followup: six years, expressed as a median). About 70% of patients with normal serum *β*_2_M did not develop the disease the next 6 years, in contrast to patients with elevated *β*_2_M, where only 35% of patients did not develop the disease for the same period. Our results show that cystatin C correlates with *β*_2_M in the total group of patients and in the group of aggressive nodal lymphoma (Table 2). When we correlate the levels of cystatin C and *β*_2_M in patients who after the third cycle of therapy experienced partial or complete remission, result is more pronounced correlation in the total sample of patients, in the group of aggressive lymphomas, and in the subgroup of aggressive nodal lymphoma (Table 2). Based on the results of correlation of cystatin C and *β*_2_M and on the basis of previous studies indicating the importance of *β*_2_M as a prognostic factor in aggressive lymphomas, it may be concluded that cystatin C could serve as an indicator of course and prognosis of the disease. 

The international prognostic index is widely accepted prognostic index for patients with aggressive lymphoma [18]. Our results show a correlation of cystatin C with IPI in the group of aggressive lymphomas and in the subgroup of aggressive nodal lymphoma (Table 4). In addition, the levels of the inhibitor were compared with three individual factors that are included in the IPI as age, serum LDH activity, and clinical stage of disease. 

In the total sample, serum cystatin C was significantly higher in patients over 60 years of age compared with those under 60 years (Table 3). Elevated levels of cystatin C in patients over 60 years of age may be the result of the weakening of kidney function due to aging [19]. However, it should be noted that all the patients involved in the study had normal serum creatinine. 

Cystatin C levels were significantly elevated in patients with elevated LDH activity compared to those with normal LDH activity in a group of aggressive lymphomas (Table 3).

In addition, our results show that cystatin C at the time of diagnosis, as well as *β*_2_M, correlates with clinical stage of disease. A statistically significant correlation was found in the group of aggressive lymphomas and in the subgroup of aggressive nodal lymphoma (Table 4). These results suggest the possibility that there is a specific mechanism in aggressive nodal lymphomas that drives increased gene expression and secretion of cystatin C in body fluids. Moreover, cystatin C is produced by all nucleated body cells, so the correlation of the inhibitor level and clinical stage of disease in aggressive nodal lymphomas can be the result of the general response of organism to the increased proteolytic activity in the infiltrated lymph nodes.

Study of Preti et al. [9] showed that IL-6 is an important independent prognostic factor in patients with diffuse large cell lymphoma, and a high value of serum interleukin 6 prior to the initiation of therapy correlates with poor response to therapy and shorter survival. However, the correlation between cystatin C and IL-6 was not determined, neither at the time of diagnosis or before the fourth cycle of chemotherapy (Table 2). 

Cystatin C shows no correlation with CRP (Table 2) which is to be expected, because the biosynthesis of CRP as an acute phase reactant in the liver is under the control of IL-6 [20]. As the inhibitor level does not correlate with the level of IL-6, there is also no correlation with CRP. In addition, the presence of B symptoms is associated with elevated inflammatory proteins including CRP [21] and cytokines, such as IL-6 [22]. Since cystatin C shows no correlation with these parameters, the result of comparison of the cystatin C levels of the group with B symptoms and of the cystatin C levels of the group without symptoms of disease is expected. 

Our results show that cystatin C should be taken into consideration in lymphoma monitoring. However, we expect that the disease-free and overall survival analysis will give the definitive answer about the reliability of cystatin C as an indicator of course of aggressive B non-Hodgkin lymphomas. This particularly refers to a group of patients with nodal disease presentation which almost completely contributes to correlation of cystatin C level and IPI. 

## Figures and Tables

**Table 1:** Comparison of cystatin C levels measured at the time of diagnosis and before IV cycle of chemotherapy in patients with partial or complete remission.

Group	No.	Median and range (ng/mL)		Wilcoxon Z	*P *
		Time of diagnosis	Before IV chemotherapy		
All patients^a^	40	944,63 (562,47–1594,30)	890,07 (618,0–1415,20)	−2,245*	0,025

Aggressive	34	944,63 (562,47–1594,30)	887,46 (618,0–1415,20)	−2,248*	0,025
Extranodal	19	931,30 (562,47–1594,30)	995,76 (698,38–1415,20)	−1,087	0,277
Nodal	15	957,96 (599,45–1451,60)	823,36 (618,0–1182,90)	−1,874	0,061

*Statistically significant difference.

^a^Total group of patients.

**Table 2:** Correlation of cystatin C with IL-6, *β*_2_M, and CRP.

Parameter	Group	Time at diagnosis			Before IV chemotherapy		
		Number of patients	Correlation coefficient	*P* value	Number of patients	Correlation coefficient	*P* value
IL-6	All patients^a^	31	0,056	0,766	26	−0,268	0,186
	Aggressive	26	0,079	0,701	20	−0,138	0,561
	Extranodal	13	0,082	0,789	11	0,345	0,298
	Nodal	13	0,110	0,721	9	−0,500	0,170

*β*_2_M	All patients^a^	50	0,332*	0,019	36	0,460**	0,005
	Aggressive	39	0,286	0,077	31	0,488**	0,005
	Extranodal	22	0,201	0,370	18	0,418	0,084
	Nodal	17	0,522*	0,032	13	0,626**	0,022

CRP	All patients^a^	52	0,107	0,451			
	Aggressive	41	0,181	0,258			
	Extranodal	23	0,381	0,073			
	Nodal	18	−0,028	0,913			

*Statistically significant correlation.

^a^Total group of patients.

**Statistically highly significant correlation.

**Table 3:** Comparisons of cystatin C levels measured at the time of diagnosis between groups of patients with normal and elevated LDH level, groups of absent and present B symptoms, and groups under and over 60 years of age.

Parameter	Number of patients			
	All patients^a^	Aggressive	Extranodal	Nodal
LDH				
Normal	33	23	13	10
Elevated	19	18	10	8
*U *	225	120*	38	21
* P* value	0,093	0,022	0,101	0,101

B symptoms				
No	12	9	6	3
Yes	40	32	17	15
*U *	150	94	29	16
* P* value	0,051	0,120	0,135	0,498

Age				
≤60 years	29	23	11	12
>60 years	22	17	11	6
*U *	191*	124	42	21
*P* value	0,015	0,051	0,243	0,180

*Statistically significant difference.

^a^Total group of patients.

**Table 4:** Correlation of cystatin C with clinical stage of the disease and IPI.

Group	No.	Clinical stage					Correlation coefficient (*r*)	*P* value
		I	II		III	IV	
All patients^a^	52	3	24		21	4	0,250	0,074
Aggressive	41	2	19		17	3	0,313*	0,046
Extranodal	23	2	12		7	2	0,242	0,265
Nodal	18	—	7		10	1	0,476*	0,046

			IPI stage					
		0	1	2	3	4		

Aggressive	41	3	13	13	7	5	0,488**	0,001
Extranodal	23	—	8	8	3	4	0,398	0,060
Nodal	18	4	4	5	4	1	0,575*	0,013

*Statistically significant correlation.

^a^Total group of patients.

**Statistically highly significant correlation.
